# Characteristics and Histological Types of Lupus Nephritis in a Jordanian Tertiary Medical Center

**DOI:** 10.1155/2019/7087461

**Published:** 2019-03-25

**Authors:** Randa I. Farah, Ebtesam Dannoun, Nisreen Abu Shahin, Saif Aldeen AlRyalat

**Affiliations:** ^1^Department of Internal Medicine, School of Medicine, The University of Jordan, Amman, Jordan; ^2^Department of Pathology and Microbiology and Forensic Medicine, School of Medicine, The University of Jordan, Amman, Jordan; ^3^Department of Ophthalmology, Faculty of Medicine, The University of Jordan, Amman, Jordan

## Abstract

**Objectives:**

Few reports of lupus nephritis (LN) from Jordan and the Middle East exist. This study assessed the demographic, clinical, and basic laboratory characteristics of Jordanian patients with LN and correlations with the histological class of LN.

**Methods:**

This was a retrospective study of all patients who underwent kidney biopsy between 2007 and 2018 at a tertiary medical center in Jordan. Patients' demographic, clinical, laboratory, and pathological data were reviewed.

**Results:**

In total, 79 patients were included in this study [mean age, 29.95 ± 12.16 years; 11 men (13.9%), 68 women (86.1%)]. Asymptomatic proteinuria and hematuria were the most common presentations in LN patients at biopsy (59.5%). The study revealed a significant difference in frequency of nephritic syndrome (p= 0.01) between sexes (10.3% female vs. 45.5% male). Class IV was the most common pathological class of LN [37 (46.8%)], followed by class V [15 (19%)] and class III [10 (12.7%)]. Post hoc analysis of the associations between laboratory values and histopathological patterns revealed a significant correlation between class IV lupus and renal failure (p= 0.018) and class IV lupus and anti-DNA antibodies p= 0.030). End-stage renal disease (ESRD) occurred in 25% of lupus nephritis cases. There was an increased likelihood of ESRD among men than women (45% vs. 22%). Overall mortality was 10%.

**Conclusion:**

Although some clinical and laboratory findings correlate with histological types of LN, clinical and laboratory parameters of Jordanian patients with LN are not predictive of the histological type, although differences with regional studies were noted.

## 1. Introduction

Systemic lupus erythematosus (SLE) is a chronic inflammatory disease with variable clinical manifestations. Lupus nephritis (LN) is one of the most common and serious manifestations of SLE [1], occurring in 40–75% of patients from various populations [2], usually within five years of disease onset, and is associated with substantial morbidity and mortality. The presentation and clinical course of LN are highly variable, ranging from benign to fulminant. Histological evidence of LN can be detected in most SLE patients, even in the absence of clinical manifestations. Histological evidence is detected via evidence of lupus activity, such as the presence of proteinuria and hematuria as well as specific immunological findings. Characteristic symptoms of LN are hypertension, proteinuria, and renal failure. Clinically relevant LN is associated with a 30% decrease in creatinine clearance, proteinuria >500 mg/dl, and renal biopsy findings indicating active LN [3]. Clinical and laboratory findings can be used to predict the histologic type of LN; however, kidney biopsy remains the cornerstone for diagnosis due to the possible toxicity of some treatment protocols and the presence of a significant correlation between histological findings, early diagnosis, and therapy [4].

In Jordan, LN is the most common biopsy-proven secondary glomerulonephritis (GN), accounting for 69% of total secondary GN cases [5], and it remains the most common secondary GN that leads to ESRD [6]. There are few current reports available from Jordan describing the demographic, clinical, and pathological features of LN and their correlations with the histological classes of LN. Previously reported data from Jordan revealed an increase in the prevalence of LN as a cause of secondary GN. As an underlying cause of secondary GN, LN was ranked fifth, responsible for 9.4% of cases in a study by Ghnaimat et al. [7]. A further study by Said et al. ranked LN as the second most common cause, responsible for 38.8% of secondary GN [8], and it was ranked the commonest cause of secondary GN in studies by Wahbeh et al. and Randa et al., responsible for 26.6% and 69% of cases, respectively [4, 9].

This study aimed to assess the demographic, clinical, and basic laboratory data of Jordanian patients with LN and correlations with the histological class of LN according to the ISN/RPS 2003 classification. Overall outcomes of SLE patients with LN in Jordan were documented. In addition, data from this study was compared with those from neighboring countries.

## 2. Methods

This retrospective study was approved by the institutional review board (IRB) of the Jordan University Hospital and was conducted in accordance with the latest update of the Helsinki Declaration. Diagnosis of SLE was based on the American College of Rheumatology (ACR) classification criteria for SLE [10]. The patients with 4 out of 11 diagnostic criteria of lupus, including at least 1 clinical and 1 immunologic criterion, and undergoing percutaneous kidney biopsy with a diagnosis of LN at the University of Jordan between January 2007 and March 2018 were included.

The data collected included baseline clinical characteristics at time of biopsy: age, sex, duration of lupus, presence of hypertension (blood pressure >140/90 mmHg), presence of renal failure (according to Acute Kidney Injury Network (AKIN) serum creatinine criteria) [11], and presence of chronic renal failure (GFR <60 ml/min/1.75 m^2^). Indications for kidney biopsy were nephrotic syndrome, nephritic syndrome, asymptomatic hematuria (defined as >5 RBCs per field), asymptomatic proteinuria (>500 mg/day), the presence or absence of other clinical abnormalities or any level of proteinuria or hematuria, and impaired kidney function that could not be attributed to another cause.

Nephrotic syndrome is defined as nephrotic-range proteinuria with a low serum albumin level and edema. Nephritic syndrome is defined with 30% increase in the serum creatinine level from baseline with elevated blood pressure.

Baseline laboratory findings were recorded, including urine analysis and 24-hour urine collection of protein or spot urine protein, to determine the urine: creatinine ratio as an evaluation of the degree of proteinuria. Other laboratory data collected included serum hemoglobin (HB); serum creatinine levels (g/dl); immunological marker levels, including anti-nuclear antibodies (ANA) (positive > 1:40) detected by indirect immunofluorescence test (IIFT) on Hep-2 cell; anti-double stranded DNA (positive at >1:20 detected by immunofluorescence assay (IFA)); serum complements C3 ( <0.9 g/l was considered low ) and C4 ( <0.1 g/l was considered low); serum albumin levels (g/dl); and serum erythrocyte sedimentation rate (ESR) (mm/hour). Anemia was defined as Hb less than 12 g/dl in females and less than 13.5 g/dl in males. All laboratory results and immunological markers were obtained at the time of kidney biopsy.

### 2.1. Statistical Analysis

All statistical analyses were performed using SPSS version 21.0 (Chicago, USA). Continuous variables (e.g., age, SLE duration, albumin level, ESR, and Hb) were presented as mean (± standard deviation), and other nominal variables (e.g., sex) were presented as number (frequency).

One-way ANOVA followed by Tukey's post hoc test was used to analyze the difference in histopathological type and the following continuous variables: age, SLE duration, albumin level, ESR, and Hb.

We also used independent-sample t-tests to analyze the mean gender differences and continues variables. Data are presented as a mean [95% confidence interval (CI)].

We used the chi-square test to analyze sex versus the following dichotomous variables: frequency of nephritic syndrome, nephrotic syndrome, proteinuria, hematuria, hypertension, C3 and C4 complements level (low or normal), ANA (positive and negative), Hb and ESR, in addition to end-stage renal disease (ESRD), or death. The above variables were also tested against histopathology type, using the chi-square test followed by the Z-test for proportions. All underlying assumptions were met, unless otherwise indicated. A* p*-value of <0.05 was considered statistically significant.

## 3. Results

A total of 79 patients were included in this study [mean age 29.95 years (±12.16)]. They included 11 men (13.9%), mean age 28.18 years (±13.88), and 68 women (86.1%), mean age 30.24 years (±11.95). The female-to-male ratio was approximately 6:1.  Table 1 details the characteristics of the patients included at the time of kidney biopsy. A total of 85.9% were ANA-positive (1:40) at time of biopsy and 40.5% were anti-DNA-positive (>1:20 by IF). Low C3 (<0.9 g/l) levels were found in approximately 75% of patients. The average time from development of systemic lupus to diagnosis of LN was 25.47±20.7 months; a diagnosis was established in 40.5% of the cases in the first 2 years (Table 1).

Asymptomatic proteinuria and hematuria were the most common presentations in LN patients (59.5%) at time of kidney biopsy, followed by nephritic syndrome (22.8%) and nephrotic syndrome (17.7%) as shown in Table 2.

The mean platelet count at time of kidney biopsy was 277,000/ *μ*l of blood and 6% of the cases were found to involve thrombocytopenia with a platelet count <140000/*μ*l of blood. The mean white blood cell (WBC) count was 7700 cells/*μ*l of blood at time of biopsy and 6.3% of cases were found to involve leukopenia at this point, with a WBC count <4000 cells/*μ*l of blood.

### 3.1. Histopathology

**Figure 1:**
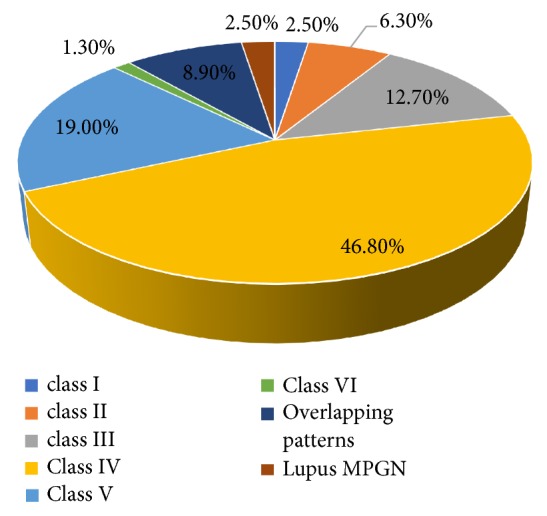
Distribution of renal pathologic findings according to the ISN/RPS 2003 classification.

The most common histopathological type of LN in this study, according to the International Society of Nephrology/Renal Pathology Society (ISN/RPS) classification, was class IV [37 (46.8%)], followed by class V [15 (19%); Figure 1] and class III [10(12.7%)]. An overlapping pattern was present in 7 cases (8.9%) (Figure 1)

Analysis of the mean difference in laboratory values and their association with histopathology type revealed that both baseline creatinine and albumin levels showed a statistically significant association with histopathology type (p= 0.013 and 0.008, respectively). On post hoc analysis, associations were only revealed with albumin levels. Analysis revealed a statistically significant association between lupus with membranoproliferative glomerulonephritis pattern (MPGN) (1.73 ±0.53) and class II (p = 0.004, albumin = 3.84 ±0.45), class III (p = 0.007, albumin = 3.62 ±0.47), class IV (p = 0.047, albumin = 3.18 ±0.67), and class V (p = 0.037, albumin = 3.28 ±0.69), respectively. We also uncovered a statistically significant association between histopathological type and renal failure (p = 0.018), with post hoc analysis showing a significant association between class IV and increased renal failure, which reached 51.4%; analysis of the other classes did not reveal statistical significance. Further analysis revealed a significant association between histopathological type and the anti-DNA level (p= 0.030), with post hoc Z-test analysis, showing that the highest level was for class IV (anti-DNA: 59.5%), and the lowest level for class V (anti-DNA: 13.3%). No statistically significant associations were found between the different histopathology types and baseline characteristics, including age; sex; proteinuria level; hematuria level; hypertension; C3, C4, and ANA level; SLE duration; ESR; and hemoglobin level at the time of diagnosis.

### 3.2. Gender

This study found a sex difference in the frequency of classes IV and V: 48.5% of female patients had class IV LN and 17% had class V LN, whereas 36.4% of male patients had class IV LN and 27.1% had class V.

There was a significant sex difference in the frequency of nephritic syndrome (p= 0.01), which was present in 10.3% of females and 45.5% of males (Table 1).

There was a significant difference between genders for levels of anemia (p= 0.004), with 85.3% of female and only 40.0% of male patients having anemia.

Results from this study indicate that the renal outcome was worse in male than in female patients (45% vs. 22% developed ESRD); however, this was not statistically significant (p= 0.103). There was no significant difference between males and females with regard to mortality (p= 0.692).

The presence of class IV LN increased the risk of developing ESRD regardless of sex, as 60% of males and 86% of females who developed ESRD had class IV lupus.

This study showed no significant difference between anti-DNA and neither mortality (p= 0.169) nor ESRD (p= 0.230). After a mean follow-up duration of 25.4 months (±20.72), the rate of mortality was 10.1% and the rate of ESRD was 25.3%.

## 4. Discussion

This study assessed the demographic, clinical, and basic laboratory characteristics of Jordanian patients with LN and their associations with the histological class of LN. The mean age of the patients in this study was 29.95 years (±12.16), and male patients were, on average, 2 years younger than female patients; this result differs from results of studies from Saudi Arabia, Kuwait, Singapore, and China, in which the mean ages of the patients were 32, 31.5, 35.4, and 33 ± 14 years, respectively [5, 12–14]. A younger age at presentation of LN is associated with a more severe form of the disease or earlier mortality [15].

This study had a male: female ratio of 1:6, which is different from that from studies in Saudi Arabia, Kuwait, Iran, and Bangladesh, where male: female ratios were 1:8.7, 1:9.8, 1:10, and 1:13, respectively [2, 5, 12, 16]. These findings are almost the same as that of a previous Jordanian study by Mustafa et al. [17] and a Singaporean study [13], with a male: female ratio of 1:4. This indicates that more male patients in Jordan have LN than in other countries (neighboring and nonneighboring). This difference may be related to specific differences in pathogenesis among sexes, including genetic, hormonal, and immune-responses factors, in addition to racial and geographical variations in LN [18]. Further studies are warranted to establish the causes of the increased prevalence of LN among males in Jordan.

In this study, the baseline clinical characteristics of LN patients were different from those of patients in previous reports because the prevalence of hypertension (74%) in our population at the time of presentation is higher than that reported by Almaani et al. (30%) [3] and in Al Arfaj et al. (43%) [5]; this can be explained by high prevalence of hypertension in Jordan as reported by Jaddou et al. [19] However, renal failure was found in only 30.4% of our patients, which was lower than that reported in Saudi Arabia (60%) [5] and by Almaani et al. (63%) [3].

The average time from development of systemic lupus to diagnosis of LN was 25.47±20.7 months. This short duration may explain the early lupus nephritis presentation in SLE patients as 75.9% of cases are identified in the first 5 years of disease. In this study, 90% of patients who developed ESRD developed renal involvement within 2 years of SLE diagnosis. As reported in previous studies, early presentation of LN in the SLE course is correlated with poor outcomes [3].

The most common indication for kidney biopsy in this study was the presence of proteinuria and hematuria (representing around 60% of the sample). These findings could support the general clinical practices of nephrologists and rheumatologists with respect to performing kidney biopsy in asymptomatic SLE patients. The prevalence of a nephrotic range of proteinuria in our cohort was approximately 34.2%, which is more than that reported in neighboring countries such as Saudi Arabia (20.6%) [5] and less than that reported in other countries as the USA (50%) [3].

We noticed a significant sex difference in the frequency of nephritis (p= 0.01), which was higher in male than in female patients, and renal outcomes, which were worse in male than in female patients (45% vs. 22% developed ESRD); however, this was not statically significant. This is consistent with results of previous reports in which increased disease severity is more often observed in male rather than in female SLE patients [20].

In this study, only 40.5% of cases with biopsy-proven LN patients had anti-DNA positivity, which differs from previous reports, suggesting that the correlation between renal involvement and anti-DNA levels could reach 90% [20]. We found a significant association between class IV LN and the anti-DNA level, which was statistically significant at 59.55%. Moreover, all patients in this cohort who developed ESRD or died were positive for anti-DNA, which suggests a strong correlation between anti-DNA and disease severity. A recent study supports the role of anti-DNA in the pathogenesis of LN and the results of this study suggest that the pathogenesis of LN may be more closely correlated with geographical and racial factors [17].

In this study, class IV was the most frequent histological type, representing approximately 46.8% of the total cases, similar to levels reported in Jordan by Mustafa et al. (60%) and observed in countries including Lebanon (38%) [21], Saudi Arabia (37.1%) [5], the UK (37%) [22], and the USA (48.6%) [23]. The second most common is class V (19.0%), which is different from that reported previously in Jordan (18%) and Saudi Arabia (18.7%), where class II was revealed to be the second most common class [5]. In countries such as the USA and the UK, class III is the second most common class [22, 23]. The increased trend in class V lupus (membranous glomerulopathy, MGN) as a second histological type of LN may be consistent with the increase in MGN in Jordan [4]. This may be related to industrialization, environmental, and behavioral changes, adding to the risk factors for development of LN in men in Jordan who are known to have higher risk of MGN.

We found there is a correlation between some clinical and laboratory findings, including renal failure, anti-DNA positivity, and class IV lupus. This can aid in prediction of the pathological pattern and in initiation of treatment. However, we could not find a correlation between other demographic, clinical, and laboratory findings and histopathological type.

In this study, 25% of patients developed ESRD and 80% had class IV lupus. This was higher than the expected risk of ESRD, which is 10% in 10 years [24]. This result could be related to many factors, including the aggressive clinical course of LN in our geographical area, inadequate use of immunosuppressive medication, loss to follow-up, late presentation, seeking treatment at other medical services, or undergoing herbal treatment. Over a mean follow-up duration of 25.4 (±20.72) months, we determined a mortality rate of 10.1% and a rate of ESRD development of 25.3%, which is considered higher than that described in previous studies [24, 25]. A higher percentage of males with LN developed ESRD, which may explain the overall poor prognosis of LN in this study.

The limitations of this study are as follows: first, although this study was performed in a tertiary referral center, it remains a single-center design. Second, the small sample size made the correlation of clinical and laboratory data with histopathological type difficult. The presence of different clinical and laboratory characteristics among our population and the poor renal outcomes emphasize the need to establish a registry and to conduct research allowing a more comprehensive interpretation of our results among different patients in order to understand the risk factors and pathogenesis of LN in our geographical area.

## 5. Conclusion

In this study, the correlations of clinical manifestations and laboratory findings with histopathologic types of LN in Jordanian patients were analyzed for the first time. We found that some demographic, clinical, and laboratory findings were different from those in previous studies of LN in the same geographical area. This study revealed correlations between clinical and laboratory findings and histopathological type. This correlation may help determine the clinical and pathologic conditions of the patient, the prognosis, and the need for immediate immunosuppressive treatment. We believe that clinical manifestations and laboratory results are helpful in the prognosis and prediction of clinical course but cannot definitively determine renal histopathology; therefore renal biopsy remains the cornerstone for identifying treatment options for LN patients. This study provides more information about LN in Jordan and future clinical research is recommended.

## Figures and Tables

**Table 1:** Demographic, clinical, and laboratory characteristics of patients.

Baseline Characteristics	Number (%)
Male patients, No (%)	11 (13.9%)

Female patients, No (%)	68 (86.1%)

Male-to-female ratio	6:1

Mean age (years)	29.95±12.16

Age range (years)	13-60 years

Hypertension, No (%)	59 (74.7%)

Hematuria, No (%)	34 (43.1%)

Proteinuria Nephrotic range	78 (98.8%) 27 (34.3%)

Renal impairments*∗∗*	(24) 30.4%

Positive-ANA (1:40)	67 (85.9%)

Positive Anti-DNA (>1:20)	32 (40.5%)

C3< 0.9 g/l	60 (75.9%)

C4 < 0.1 g/l	35 (44.3%)

Mean serum albumin (g/dl)	3.24 g/dl ±0.7 g/dl

Baseline Hb (g/dl)	10.89±1.92

Average nephritis diagnosis from lupus diagnosis (months)	25.37 months ± 20.7 months

Baseline serum creatinine (mg/dl)	1.0987 mg/dl± 0.76 mg/dl

Baseline proteinuria at time of kidney biopsy	4.26 (±4.88) g

Baseline ESR (mm/hour)	56±40

*∗∗*Renal impairments: according to Acute Kidney Injury Network (AKIN) serum creatinine criteria and presence of chronic renal failure (Glomerular filtration rate <60 ml/min/1.75 m^2^) from the Chronic Kidney Disease Epidemiology Collaboration.

**Table 2:** Clinical indication of kidney biopsy with histopathological type of lupus nephritis.

Histopathological type of LN	Nephritic Syndrome	Nephrotic Syndrome	Proteinuria and Hematuria	Total Number
Class 1% (No.)	0.0% (0)	0.0% (0)	100.0% (2)	100.0% (2)

Class II% (No.)	0.0% (0)	0.0% (0)	100.0% (5)	100.0% (5)

Class III% (No.)	0.0% (0)	0.0% (0)	100.0% (10)	100.0% (10)

Class IV% (No.)	37.8% (14)	21.6% (8)	40.5% (15)	100.0% (37)

Class V% (No.)	13.3% (2)	20.0% (3)	66.7% (10)	100.0% (15)

Class VI% (No.)	100.0% (1)	0.0% (0)	100.0% (0)	100.0% (1)

Overlapping pattern	14.3% (2)	28.6% (2)	57.1% (5)	100.0% (9)

Lupus MPGN pattern	50% (1)	0.0% (0)	50% (1)	100 (2)

Total No. (%)	19 (24.05%)	14 (17.7%)	46 (58.23%)	79 (100%)

LN: Lupus Nephritis, MPGN: membranoproliferative glomerulonephritis

## Data Availability

The data used to support the findings of this study are available from the corresponding author upon request.

## References

[1] Cervera R., Khamashta M. A., Font J. (2003). Morbidity and mortality in systemic lupus erythematosus during a 10-year period: a comparison of early and late manifestations in a cohort of 1,000 patients. *Medicine*.

[2] Nezhad S. T., Sepaskhah R. (2008). Correlation of clinical and pathological findings in patients with lupus nephritis: a five-year experience in Iran. *Saudi Journal of Kidney Disease and Transplantation*.

[3] Almaani S., Meara A., Rovin B. H. (2017). Update on lupus nephritis. *Clinical Journal of the American Society of Nephrology*.

[4] Farah R. I. (2018). Glomerulonephritis pattern at a jordanian tertiary care center. *International Journal of Nephrology*.

[5] Al Arfaj A. S., Khalil N., Al Saleh S. (2009). Lupus nephritis among 624 cases of systemic lupus erythematosus in Riyadh, Saudi Arabia. *Rheumatology International*.

[6] USRDS Annual data report 2015. http://www.usrds.org.

[7] Ghnaimat M., Akash N., El-Lozi M. (1999). Kidney biopsy in Jordan: complications and histo- pathological findings. *Saudi Journal of Kidney Disease and Transplantation*.

[8] Said R., Hamzeh Y., Tarawneh M. (2000). The spectrum of glomerulopathy in Jordan. *Saudi Journal of Kidney Disease and Transplantation*.

[9] Wahbeh A. M., Ewais M. H., Elsharif M. E. (2008). Spectrum of glomerulonephritis in adult Jordanians at Jordan university hospital. *Saudi Journal of Kidney Diseases and Transplantation*.

[10] Petri M., Orbai A. M., Alarcón et al. G. S. (2012). Derivation and validation of the systemic lupus international collaborating clinics classification criteria for systemic lupus erythematosus. *Arthritis Rheum*.

[11] Mehta R. L., Kellum J. A., Shah S. V. (2007). Acute kidney injury network: report of an initiative to improve outcomes in acute kidney injury. *Crit Care*.

[12] Jarallah K., Al-Awadi A., Siddiqui et al. H. (1998). Systemic lupus erythematosus in Kuwait - hospital based study. *Lupus*.

[13] Akhter S. (2006). *A study on histopathological patterns of lupus nephritis [Thesis]*.

[14] Gun H. C., Yoon K. H., Fong K. Y. (2002). Clinical outcomes of patients with biopsy-proven lupus nephritis in NUH. *Singapore Medical Journal*.

[15] Ong C., Nicholls K., Becker G. (2011). Ethnicity and lupus nephritis: an Australian single centre study. *Internal Medicine Journal*.

[16] Mustafa K. N., Aladily T. N., Shomaf M. S., Wahbeh A. M. (2011). Renal biopsy findings in lupus nephritis. *Saudi Journal of Kidney Disease and Transplantation*.

[17] Schwartzman-Morris J., Putterman C. (2012). Gender Differences in the Pathogenesis and Outcome of Lupus and of Lupus Nephritis. *Clinical and Developmental Immunology*.

[18] Narayanan K., Marwaha V. (2010). Correlation between systemic lupus erythematosus disease activity index, c3, c4 and anti-dsdna antibodies. *Medical Journal Armed Forces India*.

[19] Jaddou H. Y., Batieha A. M., Khader Y. S., Kanaan A. H., El-Khateeb M. S., Ajlouni K. M. (2011). Hypertension prevalence, awareness, treatment and control, and associated factors: results from a national survey, Jordan. *International Journal of Hypertension*.

[20] Goilav B., Putterman C. (2015). The role of anti-dna antibodies in the development of lupus nephritis: a complementary, or alternative, viewpoint?. *Seminars in Nephrology*.

[21] Uthman I. W., Muffarij A. A., Mudawar W. A., Nasr F. W., Masri A. (2016). Lupus nephritis in Lebanon. *Lupus*.

[22] Bono L., Cameron J. S., Hicks J. A. (1999). The very long-term prognosis and complications of lupus nephritis and treatment. *An International Journal of Medicine*.

[23] Neumann K., Wallace D. J., Azen C. (1995). Lupus in the 1980s: III. Influence of clinical variables, biopsy,and treatment on the outcome in 150 patients with lupus nephritis seen at a single center. *Seminars in Arthritis and Rheumatism*.

[24] Patrice W. (2016). *End-Stage Renal Disease Risk in Lupus Nephritis Remains Unchanged of Late Publish Date*.

[25] Singh S., Saxena R., Zhou X. J., Ahn C. (2011). A retrospective analysis of clinical presentation of lupus nephritis. *The American Journal of the Medical Sciences*.

